# Large area used by squirrel gliders in an urban area, uncovered using GPS telemetry

**DOI:** 10.1002/ece3.7644

**Published:** 2021-05-19

**Authors:** Ninon F. V. Meyer, John‐Paul King, Michael Mahony, John Clulow, Chad Beranek, Callum Reedman, Niko Balkenhol, Matt W. Hayward

**Affiliations:** ^1^ Conservation Science Research Group School of Environmental and Life Sciences The University of Newcastle Callaghan NSW Australia; ^2^ Wildlife Sciences Faculty of Forest Sciences University of Göttingen Göttingen Germany

**Keywords:** autocorrelated kernel density estimator, marsupial, movement, *Petaurus norfolcensis*, spatial ecology

## Abstract

The squirrel glider (*Petaurus norfolcensis*) is a threatened, gliding marsupial that persists in fragmented landscapes despite its restricted capacity to cross large gaps. As measures to maintain and/or restore suitable habitat depend on knowledge about the species' ecological requirements, we investigated the area used by squirrel gliders in an urban area near Newcastle, Australia. Using GPS telemetry data and the autocorrelated kernel density estimator, we estimated area used to average 10.8 ha and varied from 4.6 to 15 ha, which is equal to or greater than found in previous studies that spanned longer time periods. This has implications when identifying the minimum patch size necessary for ensuring the long‐term conservation of a squirrel glider population.

## Graphical abstract

We estimated the space use of squirrel gliders in an urban area using GPS telemetry. Area used varied from 5 to 15 ha which is in the upper range of previously reported.

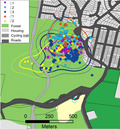

## 1 INTRODUCTION

Habitat loss and fragmentation are major drivers of species decline worldwide (Schipper et al., 2008). The squirrel glider (*Petaurus norfolcensis)* is a small, arboreal marsupial endemic to eastern Australia. The movement mode of squirrel gliders is by nonvolant gliding between trees, and they very rarely travel across the ground (Van Der Ree et al., 2004). Average glide distance of squirrel gliders is ~20 m and occasionally reaches 40–80 m (Goldingay & Taylor, 2009; Van Der Ree et al., 2004). Hence, they are highly vulnerable to canopy gaps exceeding a certain distance. The species is also threatened by the decline in abundance of large and hollow‐bearing trees that are normally used for nesting (Claridge & van der Ree, 2004).

The coast of New South Wales (NSW) has experienced substantial growth in human population in recent decades, with associated urbanization and land clearing that affect the native vegetation and fauna through fragmentation (reduction in size and isolation of forest patches; Smith & Murray, 2003). To mitigate these effects, various habitat restorations and enhancements (e.g., via gap closing poles) have been implemented in some impacted systems to maintain or expand available squirrel glider habitat and improve connectivity to ensure the long‐term survival of the species. Practitioners also often recommend a certain amount of habitat to retain to enhance squirrel glider conservation when an area is developed.

The success of such efforts largely relies on a detailed understanding of the spatial requirements of the species (Goldingay, 2015), in particular the area traversed by an individual for food gathering, mating, and caring for young, Burt's (1943) definition of home range. The spatial ecology of squirrel gliders has previously been investigated using very high frequency (VHF) telemetry (e.g., Sharpe & Goldingay, 2007; Van Der Ree & Bennett, 2003). Recent improvements in GPS tracking technology have led to smaller tracking devices that are suitable for smaller animals, while providing high‐resolution spatial data for several individuals simultaneously (Kays et al., 2015). We therefore used GPS technology to investigate the area used by squirrel gliders in an urban area of NSW.

## 2 METHODS

### 2.1 Study area

**FIGURE 1:**
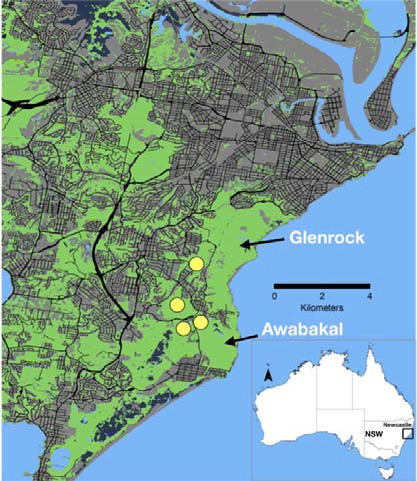
Forest cover (green) and urban area (grey) near Newcastle, NSW on the eastern coast of Australia. We trapped squirrel gliders in four patches (yellow), near the Awabakal Reserve and the Glenrock State Conservation Area

The suburban area south of Newcastle is a mosaic of forest patches that are embedded within a matrix of mainly residential and other urban developments (Figure 1), many of which are managed by the Lake Macquarie City Council (LMCC). Around 25% of remaining habitat suitable for squirrel gliders in the area will potentially be affected by development by 2030 (Fallding, 2015). Despite the high level of urbanization, populations of squirrel gliders persist in the area. In particular, some of the undeveloped patches harboring squirrel gliders lie between the Glenrock State Conservation Area (533 ha) and the Awabakal Nature Reserve (227 ha), both identified as core areas for the species (Fallding, 2015). Hence, maintaining functional connectivity is important for the long‐term conservation of the species in the area. Specifically, we surveyed four patches (35–86 ha; Figure 1) of dry sclerophyll forest and with *Banksia spp*. understory that provide flowering resources throughout the year. As squirrel gliders feed primarily upon nectar and pollen (and occasionally sap, gum, and insects), these patches were suitable habitat for them. The vegetation communities in the patches, along with dominant canopy and shrub species, included spotted gum forest (*Corymbia maculata, Eucalyptus paniculata, Dodonaea triquetra*), snappy gum forest (*C*. *gummifera, Angophara costata, E. piperita, Allocasuarina littoralis*), and coastal sheltered apple—peppermint forest (*E. piperita, E. punctata, A. costata, Melaleuca linariifolia*). Logging for mining props ceased before the 1940s, while mining activities ceased in the 1980s. Despite bushfires in the area in 2012 and 2013, the primary canopy species have a diverse age‐class structure with many suitable hollows for squirrel gliders to nest.

### 2.2 Squirrel glider data

We captured squirrel gliders in March–April 2020 by placing 8–15 pipe traps (Winning & King, 2007) per patch that we checked every morning at dawn. The captured squirrel gliders were sexed, weighed, and ear‐tagged (if not done previously), and age was estimated via teeth wear and patagium color (Quin, 1995). Adults were fitted with a transmitter that had both store‐on‐board GPS and VHF (PinPoint GPS‐Beacon 75/120, Lotek, UK) attached with a collar and weighing less than 5 g (i.e., 2.5%–3.3% of body mass of squirrel gliders). Animals were released onto trees at the point of capture and followed until they reached their den tree. We monitored the tagged animals and identified their den‐tree location every morning for 1 week until traps were reactivated to retrieve the GPS tags with the stored data.

The battery life of the GPS tags was limited due to small size of squirrel gliders, so we made a trade‐off between the fix schedule and the number of days the tags would be working. Since our original research question was to investigate fine‐scale movement pattern, and because the squirrel gliders are nocturnal, we programmed the tags to record the GPS locations every 15 min for 8 hr from dusk on (~6:30 p.m., AEST) during 6 days. The VHF schedule was from 7 to 9 a.m. to record the den tree daily. We did not discard the fixes following the captures from our analysis because the individuals had the whole day to recover before going out at night when their locations were recorded. Moreover, the use of GPS tags does not require the presence of people to record the positions, and which could alter movement patterns of the animals.

Nontarget species were released immediately after identification (i.e., brown antechinus *Antechinus stuartii*, feathertail glider *Acrobates pygmaeus*, and bush rat *Rattus fuscipes*). All procedures followed standard protocols approved by the Animal Care and Ethics Committee of the University of Newcastle (A‐2015‐510) and NSW Scientific License SL100190. LMCC granted permission to work on land managed by Council.

### 2.3 Data analysis and used area

We used the semivariance approach developed by Fleming et al. (2014) and followed the workflow described in Calabrese et al. (2016) to generate continuous‐space and continuous‐time stochastic movement models prior to estimating the used area. While there is no perfect estimator (Signer & Fieberg, 2021), we favored this method over more conventional frameworks because these models accommodate autocorrelated data with uneven sampling intervals, gaps (frequent when using GPS transmitters under the canopy), and short periods of data collection, and can estimate individual home ranges with greater accuracy (Noonan et al., 2019). This approach thus decreases the likelihood of underestimating area that is a common issue when using nonparametric methods with autocorrelated data, and which assumes that data are independently and identically distributed (Fleming et al., 2015; Noonan et al., 2019).

We initially examined whether squirrel gliders displayed range residency by inspecting their individual empirical semivariogram, which is a plot of the semivariance in relocations as a function of the time lag among observations. More specifically, this measures the distance between two relocations and computes the variability of distances among all relocation pairs with the same time lag (Fleming et al., 2014, 2015). The plots displayed an asymptote, indicating range residency of all the squirrel gliders. We then fitted three candidate models that featured a home range with the *ctmm* R package and ranked them based on the AICc (detailed in Calabrese et al., 2016; Table 1). After selecting the appropriate model, we estimated the utilization area conditional to that model with the autocorrelated kernel density estimator (AKDE) and examined overlap between the individuals' utilization areas (based on the intensity‐of‐use; Winner et al., 2018). We also calculated the minimum convex polygon (MCP) and the kernel density estimation (KDE) with the R package *amt* (Signer et al., 2018) to allow comparison with previous studies.

**TABLE 1:** Weight, movement models (AICc), and size of used area (with their confidence intervals) of five squirrel gliders near Newcastle, NSW

	♀1	♀2	♀3	♂1	♂2
Weight (g)	152	177	175	180	200
No. of relocations	86	79	106	18	28
Duration (nights)	4.1	4.6	4.7	2	1.6
Movement model					
OU	0	0	0	0	0
OUF	2.3	2.3	2.2	4.1	1.1
IID	82.3	35.0	232.1	15.5	34.6
Used area (ha)					
AKDE	4.6 (3.2–6.4)	9.8 (6.9–13.1)	15.0 (9.1–22.4)	10.7 (4.6–19.5)	10.8 (4.2–20.4)
KDE 95%	4.3 (3.5–5.3)	9.0 (7.1–11.1)	11.2 (8.3–14.7)	8.9 (5.2–13.7)	7.6 (3.9–12.5)
MCP 100%	4.9	13.8	11.3	5.1	5.4

Note

Used areas were estimated using AKDE, KDE with 95% isopleths, and 100% MCP.

Abbreviations: AKDE, autocorrelated kernel density estimation; IID, independent identically distributed process; KDE, kernel density estimation; MCP, minimum convex polygon; OU, Ornstein–Uhlenbeck process (Brownian motion within a home range, i.e., random, undirected movement); OUF, Ornstein–Uhlenbeck motion model with foraging included.

## 3 RESULTS AND DISCUSSION

We captured a total of 15 individuals over a period of 376 trapping nights, and fitted a GPS tag to 11 individuals in 2 patches. We could only analyze the spatial data of 5 individuals (3 females, 2 males) from a single patch due to failing tags. On average, the tags recorded 63.4 fixes (ranging from 18 to 106 fixes per animal; average successful fixing rate of 55%, ranging from 28% to 70%) during 3.4 nights (ranging from 1.6 to 4.7 nights; Table 1). We refer to area used instead of home range because of this short duration.

**FIGURE 2:**
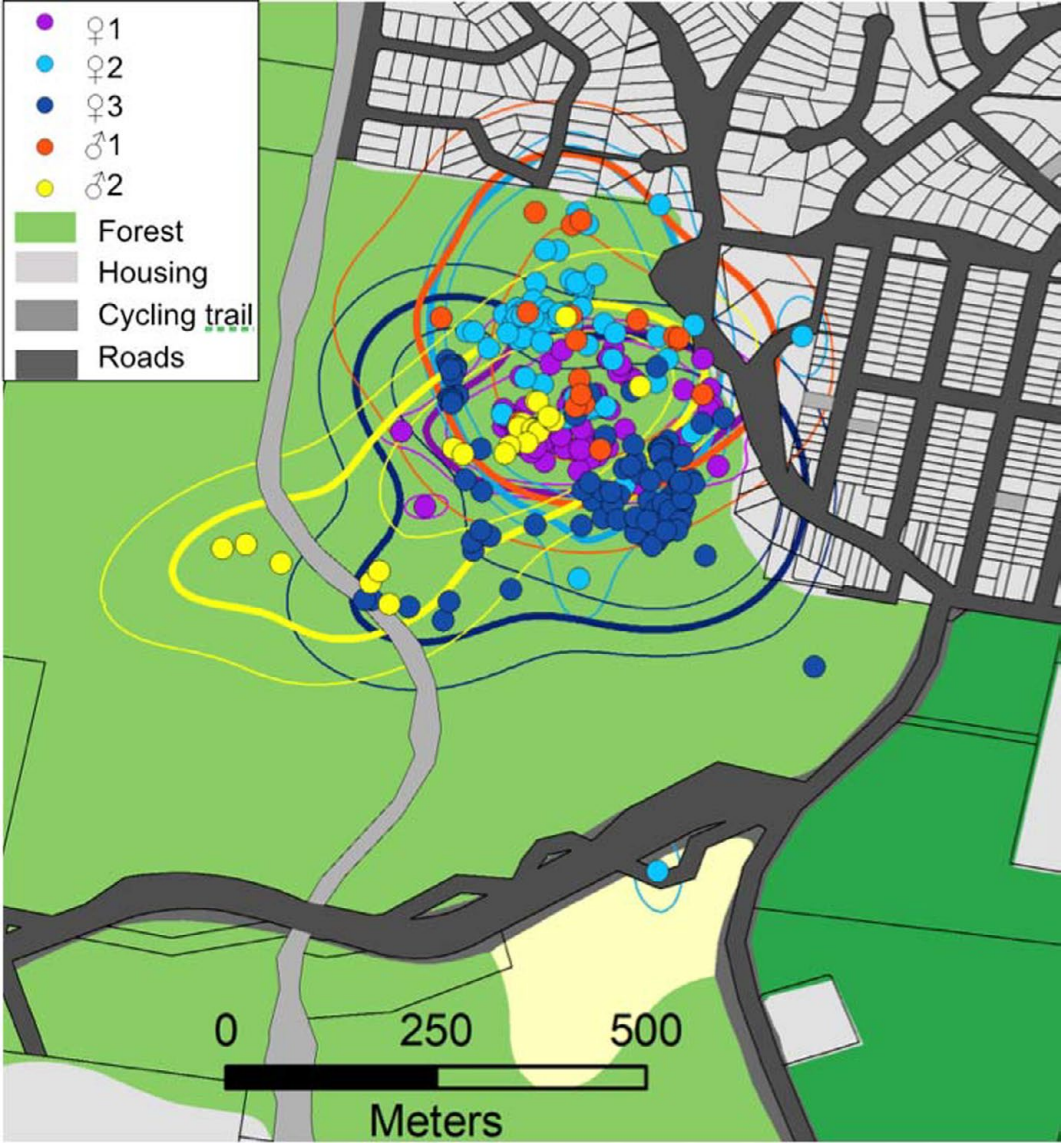
Relocations and area used determined with the autocorrelated density estimator (bold contour lines) with their 95% confidence intervals (light contour lines) of five squirrel gliders in an urban area in NSW

The Ornstein–Uhlenbeck (OU) process that combines Brownian motion (i.e., regular diffusion) with a tendency to remain in a particular area was the model that best supported the tracking data of each glider (Table 1). Conditioned on the best supported movement model, the average AKDE area used estimation was 10.8 ha and varied from 4.6 to 15 ha (Table 1). All the used areas overlapped considerably (pairwise overlap indices varied from 0.55 to 0.96), even between members from apparent distinct social groups (e.g., M1 and M2 on Figure 2), and there was no significant difference between sexes as suggested by the overlapping confidence intervals (Table 1; Figure 2). When using other indices, the estimates of area used varied from 4.3 to 11.2 ha (KDE 95%) and 4.9 to 13.8 ha (MCP %).

Our estimates are conservative given that they are based on less than a week‐long data collection period. Yet, with a few exceptions (Goldingay et al., 2010) and regardless of the estimator we used, they are equally large or larger than home ranges reported in other studies conducted in contiguous or fragmented habitats (Table 2). This is underscored by our relatively large confidence intervals which lower estimates (AKDE, ranging from 3.2 to 9.1, average of 5.6 ha; KDE, ranging from 3.5 to 8.3 ha, average of 5.6 ha; Table 1) are still larger than the average home range estimates of all other studies but one in Tea Gardens, NSW (Goldingay et al., 2010). Furthermore, our estimates of MCP are larger than those reported in other studies. Eastern Australia has recently experienced severe drought, including in late summer/early autumn 2020 when this research took place, that has affected flora and especially the flowering of eucalyptus species (Maslen, 2018). The patchy distribution of resources has likely contributed to our results, as squirrel gliders would have to forage over larger areas to fulfill their daily requirements (also reported in Goldingay et al., 2010).

**TABLE 2:** Home range (HR) estimates (with range of values when available, or ±*SE*), number of squirrel gliders tracked, and length of tracking period in several sites and types of habitat across the species range in eastern Australia (studies are ordered chronologically)

Site	Mean home range (ha)	Estimator	No. of gliders	Tracking period	Source
Chiltern, VIC	13–14	MCP	11	–	Trail (1994)
Limeburners Creek, NSW *Nature Reserve*	3.1 (0.7–8.6)	HMM^1^ 80%	40	Long‐term HR	Quin (1995)
	1.3 (0.8–1.7)	Added‐squares		Seasonal HR	
Eleebana, NSW *Suburban*	7.6 (3.9–10.1)	MCP 100%	4	16–19 nights within 3 months	SWC Consultancy (1996)
Euroa, VIC *Agricultural landscapes*	2.6 (0.7–4.8)	Grid cell estimator 95%	20	Summer (mean = 40 days)	Van Der Ree and Bennett (2003)
	2.2 (1.2–6.2)		19	Autumn (mean = 34 days)	
	2.1 (1.1–3.3)		6	Winter (mean = 19 days)	
	1.7 (0.9–2.5)		6	Spring (mean = 18 days)	
	3.9 (2.2–5.4)		4	4 seasons	
Bungawalbin, NSW *Nature Reserve*	6.2 (± 0.6)	AK^2^ 95%	9	5–62 nights (mean = 36.4 nights)	Sharpe and Goldingay (2007)
	2.8 (± 0.6)	HM 80%			
	5.6 (± 0.6)	MCP 100%			
Minnippi Parklands, QLD *Suburban*	4.6 (± 0.7)	FK^3^ 95%	12	0.8–5 months (mean = 3.5 months and 43 locations/individual)	Goldingay et al. (2010)
	6.7 (± 1.5)	MCP 100%			
Tea Gardens, NSW *Suburban*	14.8 (± 2.4)	FK 95%	6	0.2–1.1 months (mean = 1 month and 42 locations/individual)	Goldingay et al. (2010)
	13.3 (± 3.1)	MCP 100%			
South‐east Queensland *Suburban*	4.8 (2.1–8.7)	FK 95%	30	3–8 months	Brearley et al. (2011)
	3.9 (2.2–5.4)	MCP 100%			
South West Slopes, NSW *Agricultural landscapes*	4.9 (2.5–12)	Grid cell estimator	32	4–5 months (mean of 21 and 12 diurnal and nocturnal locations, respectively)	Crane et al. (2014)
Newcastle, NSW *Suburban*	10.8 (4.6–15)	AKDE	5	5 nights	This study
	8.2 (4.3–11.2)	KDE 95%			
	8.1 (4.9–13.8)	MCP 100%			

Abbreviations: QLD, Queensland; VIC, Victoria.

^1^ HMM = harmonic mean measure.

^2^ AK = adaptive kernel.

^3^ FK = fixed Kernel.

While gliders showed short‐term patterns of stable space use, as evidenced by the range residency detected by our analysis, it is likely that they did not reveal the full extent of the area required to satisfy their needs (reproductive, food, and shelter) throughout the year. We expect the area they use to increase even more with additional fixes collected over a longer period than one season (e.g., squirrel gliders home range sizes stabilized after ~4 months in southern NSW; Crane et al., 2014).

In view of our findings, this study raises two important questions: (a) is the area used by squirrel gliders in our study area unusually large? or (b) are the home ranges in other studies underestimated because of the methodology and/or technology used (also questioned by Goldingay et al., 2010)? Either way, our results have direct implications for re‐examining the current conservation strategies and informing strategic land use planning and management. For example, practitioners in our areas often use a minimum patch size of 4 ha to prioritize areas for squirrel glider conservation or for assessing the impact of development plans (Fallding, 2015). Our study suggests that re‐evaluating and increasing the minimum patch threshold to 10 ha is necessary for the long‐term conservation of this threatened species. While smaller patches can be used as stepping stones for dispersal movement or colonization of a new area, squirrel gliders usually live within family groups meaning the area used by the group is generally larger than individual home range (Sharpe & Goldingay, 2007). As a result, an even larger size of patches may be required to sustain a resident population and achieve minimum viable population sizes.

Safeguarding sufficiently large areas is not enough as the conservation of squirrel gliders also depends on the retention of a mosaic of habitats to ensure seasonal continuity of food resources, in particular winter and spring flowering trees (Sharpe & Goldingay, 2007). Despite a relatively high mobility capacity, squirrel gliders often display a high site fidelity and rarely shift range with seasonal variation (but see Van Der Ree & Bennett, 2003). Further exploring the varying pattern in resources use over several seasons with accurate GPS tags will help to understand the constraining role of forage availability and habitat quality in driving the species use of area. This is relevant to develop a more complete picture of the temporal‐spatial habitat requirements of squirrel gliders (Goldingay, 2015).

Finally, squirrel gliders tended to remain in the forest patch but occasionally crossed over a cycling trail (*n* = 6) and a 2‐lane road ~20 m wide (*n* = 2; Figure 2). Our observations concur with Brearley et al. (2011) who found that squirrel gliders use residential urban edges as habitat and can cross two‐lane roads if there are tall trees on either side. Given gap width is a limiting factor to movement and dispersal, these findings highlight the importance of the strategic retention and planting of squirrel glider food trees in urban areas. This may be especially beneficial in our study region where there is an increased demand for urban development likely to further fragment suitable habitats (Fallding, 2015).

## CONFLICT OF INTEREST

None declared.

### AUTHOR CONTRIBUTIONS

**Ninon F. V. Meyer:** Conceptualization (lead); data curation (lead); formal analysis (lead); funding acquisition (lead); investigation (lead); methodology (lead); project administration (lead); resources (equal); writing‐original draft (lead). **John‐Paul King:** Conceptualization (supporting); investigation (supporting); validation (equal); writing‐review & editing (supporting). **Michael Mahony:** Funding acquisition (supporting); writing‐review & editing (supporting). **John Clulow:** Project administration (supporting); visualization (supporting); writing‐review & editing (supporting). **Chad Beranek:** Data curation (supporting); writing‐review & editing (supporting). **Callum Reedman:** Data curation (supporting); writing‐review & editing (supporting). **Niko Balkenhol:** Conceptualization (supporting); funding acquisition (supporting); supervision (supporting); writing‐review & editing (supporting). **Matt W. Hayward:** Conceptualization (supporting); funding acquisition (supporting); supervision (supporting); writing‐review & editing (supporting).

## Data Availability

Data were uploaded on Movebank (movebank.org; https://www.movebank.org/cms/webapp?gwt_fragment=page=studies,path=study1098350542).
